# ZnCl_2_ Mediated Synthesis of InAs Nanocrystals with Aminoarsine

**DOI:** 10.1021/jacs.2c02994

**Published:** 2022-06-01

**Authors:** Dongxu Zhu, Fulvio Bellato, Houman Bahmani Jalali, Francesco Di Stasio, Mirko Prato, Yurii P. Ivanov, Giorgio Divitini, Ivan Infante, Luca De Trizio, Liberato Manna

**Affiliations:** ^†^Nanochemistry, ^‡^Photonic Nanomaterials, ^§^Materials Characterization, and ^∥^Electron Spectroscopy and Nanoscopy, Istituto Italiano di Tecnologia, Via Morego 30, 16163 Genova, Italy; ⊥ Dipartimento di Chimica e Chimica Industriale, Università di Genova, 16146 Genova, Italy

## Abstract

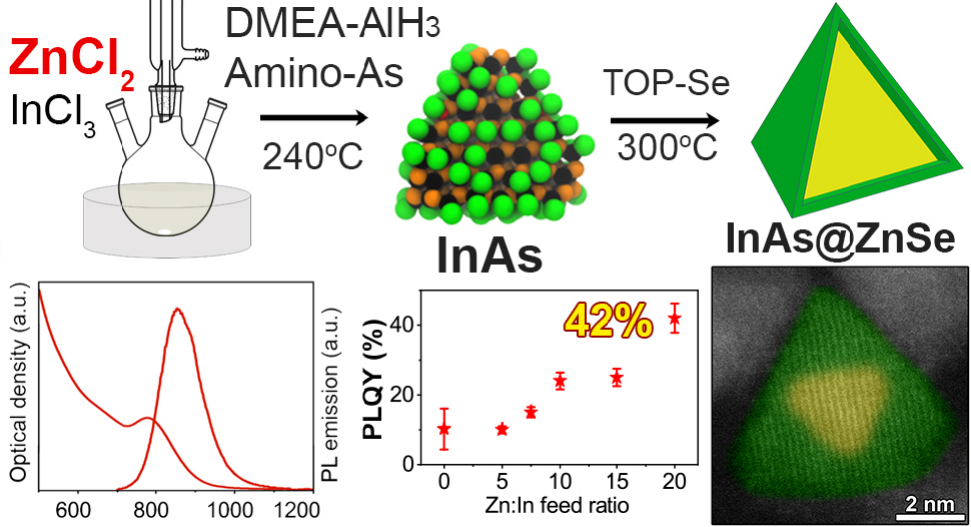

The most developed approaches for the synthesis of InAs nanocrystals (NCs) rely on pyrophoric, toxic, and not readily available tris-trimethylsilyl (or tris-trimethylgermil) arsine precursors. Less toxic and commercially available chemicals, such as tris(dimethylamino)arsine, have recently emerged as alternative As precursors. Nevertheless, InAs NCs made with such compounds need to be further optimized in terms of size distribution and optical properties in order to meet the standard reached with tris-trimethylsilyl arsine. To this aim, in this work we investigated the role of ZnCl_2_ used as an additive in the synthesis of InAs NCs with tris(dimethylamino)arsine and alane *N*,*N*-dimethylethylamine as the reducing agent. We discovered that ZnCl_2_ helps not only to improve the size distribution of InAs NCs but also to passivate their surface acting as a Z-type ligand. The presence of ZnCl_2_ on the surface of the NCs and the excess of Zn precursor used in the synthesis enable the subsequent *in situ* growth of a ZnSe shell, which is realized by simply adding the Se precursor to the crude reaction mixture. The resulting InAs@ZnSe core@shell NCs exhibit photoluminescence emission at ∼860 nm with a quantum yield as high as 42±4%, which is a record for such heterostructures, given the relatively high mismatch (6%) between InAs and ZnSe. Such bright emission was ascribed to the formation, under our peculiar reaction conditions, of an In–Zn–Se intermediate layer between the core and the shell, as indicated by X-ray photoelectron spectroscopy and elemental analyses, which helps to release the strain between the two materials.

## Introduction

Semiconductor nanocrystals (NCs) emitting in the infrared spectral region are very appealing building blocks for applications ranging from telecommunications^1^ to night vision,^2^ photovoltaics,^3,4^ lasing,^5^ and *in vivo* biological imaging.^6,7^ To date, the most studied near-infrared (NIR) and short-wave infrared emitting NC compounds are Hg-based (II–VI) or Pb-based (IV–VI) semiconductors, whose synthesis and optical properties have been optimized over the past decades.^8−10^ The presence of Hg or Pb, however, severely restricts the use of such compounds in commercial applications due to the EU’s “Restriction of Hazardous Substances” (RoHS) directives, prompting the search for alternative lead- and mercury-free RoHS compliant materials.^11−16^ Among the possible candidates, III–V semiconductors, in particular InAs NCs, have been indicated as the most appealing alternatives.^17,18^ One important reason is that the absorption and photoluminescence (PL) of InAs NCs can be tuned from 750 to 1400 nm, thus covering a large fraction of the NIR range with a single material. However, the synthesis of InAs is far more complex than that of the more studied II–VI and IV–VI NCs. This is mostly due to two reasons: (i) the very restricted choice of suitable As precursors and (ii) the covalency of the In–As lattice, which entails a poor crystallinity of the NCs under the growth conditions of colloidal synthesis approaches.^19^

The most developed synthesis route of colloidal InAs NCs relies on the use of tris-trimethylsilyl arsine (TMS-As) or its “analogues”, such as tris-trimethylgermil arsine (TMGe-As). These compounds are highly reactive, pyrophoric, toxic, and actually very hard to purchase.^18,20−29^ With the aim of substituting TMS-As/TMGe-As with less toxic, cheaper, and commercially available As precursors, Grigel et al. developed in 2016 the first synthesis of InAs NCs by employing tris(dimethylamino)arsine (amino-As).^30^ Their approach is based on the hot-injection of a reducing agent, that is, tris(dimethylamino)phosphine (amino-P), into a solution of InCl_3_ in oleylamine and amino-As. The role of the reducing agent is critical as it enables the As^3+^ → As^3–^ reduction and, thus, the regulation of the kinetics of nucleation and growth of InAs NCs.^30−32^ After the work of Grigel et al., several other reductants have been explored with the purpose to optimize the control over the size distribution of the NCs.^31−35^ Of particular interest is the work of Srivastava et al., who systematically tested and compared several reducing agents, namely, amino-P, diisobutylaluminum hydride (DIBAL-H), LiEt_3_BH, and alane *N*,*N*-dimethylethylamine (DMEA-AlH_3_),^35^ the latter enabling the best control over the size distribution of the InAs NCs.

The reports discussed above highlight how substantial progress has been made in refining the synthesis of InAs NCs with amino-As. Yet, improvements are still needed to meet the standards reached with the TMS-As/TMGe-As precursors. For example, the width of the first exciton absorption peak (normally measured as half width at half-maximum, HWHM) of InAs NCs, which is strictly correlated to the size distribution of the sample, can be as narrow as 60 meV ^28^ or even 40 meV ^36^ when employing TMS-As/TMGe-As, while in the case of amino-As the best value reported so far is around 100 meV.^34,35^ Additionally, the PL quantum yields (QYs) characterizing Cd-free InAs-based core@shell NCs made with TMS-As/TMGe-As, such as InAs@InP@ZnSe NCs, can be as high as 23%, while those synthesized with amino-As (i.e., InAs@ZnSe or InAs@ZnS) could only reach ∼10%.^14,26,30,31^

It was noted that ZnCl_2_ has been extensively used as an additive in the synthesis of InAs NCs based on amino-As and amino-P, the latter acting as a mild reducing agent. ZnCl_2_ is actually believed to trigger the NCs formation by activating the amino-P reducing agent, which would not work otherwise.^30,31^ On the other hand, ZnCl_2_ has not been employed in synthesis routes based on reducing agents stronger than amino-P, for example, DIBAL-H and DMEA-AlH_3_, as it was probably deemed as an unnecessary additive in such cases. Yet, we hypothesized that even when using such stronger reducing agents, the presence of ZnCl_2_ would be beneficial, as it could regulate the nucleation/growth of InAs NCs via passivation of their surface or even by forming In–Zn–As alloys.

**Scheme 1:**
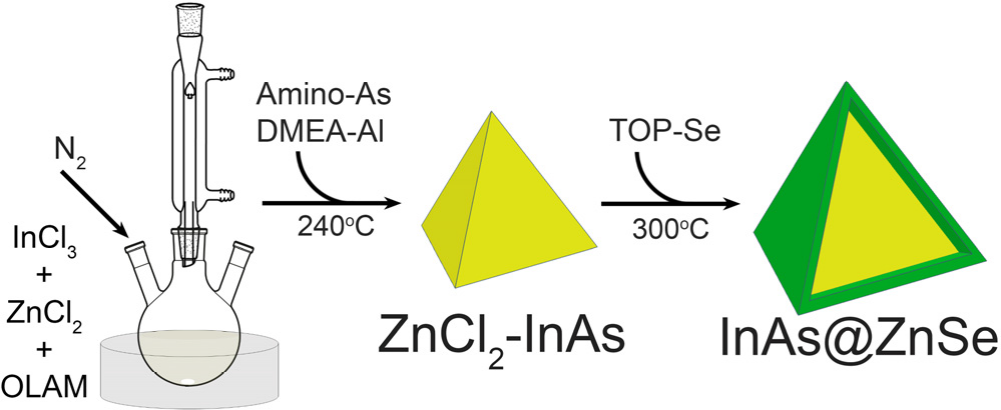
Synthesis of InAs NCs with ZnCl_2_ and Subsequent Overgrowth of a ZnSe Shell

In order to test such hypothesis and to study the effects of ZnCl_2_, in this work we have performed the synthesis of InAs NCs by combining amino-As with the best reducing agent found so far, DMEA-AlH_3_,^35^ and using variable amounts of ZnCl_2_ (Scheme 1). Our experimental results indicate that the presence of such Zn salt entails three beneficial effects: (i) an improvement of the InAs NCs size distribution, as revealed by the narrowing of the absorption exciton peak, whose HWHM was reduced from ∼130 meV (in the absence of Zn) to ∼120 meV (with NCs mean size of 2.8 ± 0.2 nm); (ii) a surface Zn incorporation, in the form of ZnCl_2_ and an improvement of the PL of the NCs, with PLQY increasing from <1% to over 2% as a result of surface traps passivation; (iii) perhaps even more remarkable than effects i and ii, the presence of Zn on the surface of the InAs NCs and the residual Zn precursor in the reaction batch could easily promote the *in situ* growth of a ZnSe shell (via the injection of the Se precursor into the crude reaction solution, Scheme 1) leading to InAs@ZnSe core@shell NCs with PLQY values as high as 42±4% (with emission at ∼860 nm). This result, which sets a new record in the PL efficiency of InAs@ZnSe NCs made by amino-As, is quite surprising as the lattice mismatch between InAs and ZnSe is around 6%, and this high value commonly leads to strained core@shell structures.^25,30,31^ Instead, in our peculiar *in situ* experimental conditions, the formation of an intermediate In–Zn–Se layer between the InAs core and the ZnSe shell was observed, which is believed to reduce the lattice strain. Our work narrows the gap between InAs NCs made with “greener” amino-As and those synthesized with the more toxic, expensive, and pyrophoric TMS-As/TMGe-As precursors, paving the way to further improvements in the synthesis of this technologically important type of NC.

## Experimental Section

### Materials

Materials were indium(III) chloride (InCl_3_, 99.999%, Sigma-Aldrich), zinc(II) chloride (ZnCl_2_, 99.999%, Sigma-Aldrich), tris(dimethylamino)arsine (amino-As, 99%, Strem), alane *N*,*N*-dimethylethylamine complex solution (DMEA–AlH_3_, 0.5 M solution in toluene, Sigma-Aldrich), selenium powder (Se, 99.99%, Strem), triethyloxonium tetrafluoroborate (Et_3_OBF_4_, 97%, Sigma-Aldrich), oleylamine (OLAM, 98%, Sigma-Aldrich), tri-*n*-octylphosphine (TOP, 97%, Strem), toluene (anhydrous, 99.8%, Sigma-Aldrich), ethanol (anhydrous, 99.8%, Sigma-Aldrich), hexane (anhydrous, 95%, Sigma-Aldrich), and *N*,*N*-dimethylformamide (DMF, anhydrous, 99.8%, Sigma-Aldrich). All the chemicals were used without further purification.

### Preparation of the As Precursor

The As precursor was prepared following a previously reported method by Srivastava et al.^32,35^ In a N_2_ filled glovebox, 0.2 mmol of amino-As was dissolved in 0.5 mL of degassed oleylamine at 40 °C for 5 min until no bubbles further evolved.

### Preparation of 1 M TOP-Se Precursor

In a N_2_ filled glovebox, 10 mmol of Se powder was mixed with 10 mL of TOP in a 20 mL glass vial and heated at 150 °C under constant stirring for ∼10 min to form a transparent solution, and then the mixture was cooled down to room temperature.

### Synthesis of InAs and ZnCl_2_-InAs Nanocrystals

In a typical synthesis, 0.2 mmol of InCl_3_, variable amounts (see below) of ZnCl_2_, and 5 mL of oleylamine were loaded into a 100 mL three-necked flask under an inert atmosphere and dried at 120 °C under vacuum for 1 h. The mixture was heated to 240 °C. The As precursor was then injected into the flask, quickly followed by the injection of 1.2 mL of the DMEA-AlH_3_ toluene solution. The reaction was carried out for 15 min and was then quenched by removing the heating mantle. When the reaction mixture reached a temperature of 90 °C, it was transferred into a nitrogen filled glovebox. The NCs were washed by the addition of toluene and ethanol and precipitated by centrifugation at 4500 rpm for 5 min. The precipitate was dispersed in toluene and centrifuged at 5500 rpm for 5 min to remove the insoluble byproducts. The supernatant was precipitated by the addition of ethanol followed by centrifugation at 4500 rpm for 5 min. The final product was dispersed in 6 mL of toluene for further characterizations. All the purification steps were carried out under N_2_ atmosphere. Several samples were prepared by systematically varying the ZnCl_2_:InCl_3_ precursors ratio from 0:1 to 5:1, 7.5:1, 10:1, 15:1, and 20:1.

### Synthesis of InAs@ZnSe Core@shell Nanocrystals

After quenching the growth of the InAs NCs by cooling the reaction mixture to 90 °C, 1 mL of 1 M TOP-Se (1 mmol of Se, Se:In ratio of 5:1) precursor was injected into the flask, and the corresponding mixture was heated up (heating rate of ∼30 °C/min) to 300 °C for 15 min. The reaction mixture was transferred into a vial under N_2_ by a glass syringe to quench the reaction. The core@shell NCs were purified by dispersion in toluene and by precipitation with ethanol. The final product was dispersed in toluene and stored in the glovebox. In the case of InAs NCs grown in the absence of ZnCl_2_, three different shelling strategies were performed which are described in the Supporting Information.

### Ligand Stripping Procedure

The ligand stripping was performed by following the procedure reported by Rosen et al.^37^ In a N_2_ glovebox, 0.5 mL of the NCs dispersion in toluene was added to 1 mL of hexane in a glass vial, and then 1 mL of a solution of Et_3_OBF_4_ in DMF (100 mM) was added into the vial. After shaking the vial for several seconds, the NCs were transferred from the hexane into the DMF phase. The NCs dispersed in DMF were precipitated by the addition of toluene followed by centrifugation at 4000 rpm for 5 min. To remove residual organic ligands, the washing procedure was repeated twice and the resulting NCs were dispersed in DMF.

### X-ray Diffraction (XRD)

XRD analysis was performed on a PANanalytical Empyrean X-ray diffractometer, equipped with a 1.8 kW Cu Kα ceramic X-ray tube and a PIXcel3D 2 × 2 area detector, operating at 45 kV and 40 mA. Concentrated NC solutions were drop-cast on a zero-diffraction single crystal substrate in glovebox and then collected under ambient conditions and room temperature. XRD data were analyzed by the HighScore 4.1 software from PANalytical.

### Transmission Electron Microscopy (TEM) Characterization

Diluted NC solutions were drop-cast onto copper TEM grids with an ultrathin carbon film. Low-resolution TEM images were acquired on a JEOL JEM-1400Plus microscope with a thermionic gun (W filament) operated at an acceleration voltage of 120 kV. High resolution scanning transmission electron microscopy (STEM) images were acquired on a probe-corrected ThermoFisher Spectra 300 S/TEM operated at 300 kV. Images were acquired on a high-angle annular dark field (HAADF) detector with a current of 150 pA. Compositional maps were acquired using Velox, with a probe current of ∼300 pA and rapid rastered scanning. The energy-dispersive X-ray (EDX) signal was acquired on a Dual-X system comprising two detectors on either side of the sample, for a total acquisition angle of 1.76 sr. Elemental maps were produced after rebinning and denoising using principal component analysis within Hyperspy.^38,39^

### Scanning Electron Microscopy (SEM)

SEM analysis was carried out on a HRSEM JEOL JSM-7500LA microscope with a cold field-emission gun (FEG) operating at 15 kV acceleration voltage. EDX spectroscopy (Oxford Instruments, X-Max, 80 mm^2^) was used to evaluate the elemental composition of the samples.

### X-ray Photoelectron Spectroscopy (XPS)

Specimens for XPS were prepared from concentrated NC solutions, dropped on freshly cleaved highly oriented pyrolytic graphite substrates in a glovebox. XPS measurements were carried out on a Kratos Axis UltraDLD spectrometer using a monochromatic Al Kα source, operated at 20 mA and 15 kV. High resolution analyses were carried out at a pass energy of 10 eV. The Kratos charge neutralizer system was used on all specimens. Spectra were charge-corrected to the main line of the carbon 1s spectrum (adventitious carbon) set to 284.8 eV. Spectra were analyzed using CasaXPS software (version 2.3.24).

### Inductively Coupled Plasma (ICP)

The elemental analysis was also performed via inductively coupled plasma optical emission spectroscopy (ICP-OES) with an iCAP 6300 DUO ICP-OES spectrometer (ThermoScientific). The samples were dissolved in 1 mL of aqua regia (HCl/HNO_3_ = 3/1 (v/v)) overnight and then diluted with 9 mL of Milli-Q water for measurements. The elemental analysis using ICP-OES was affected by a systematic error of ∼5%.

### Optical Measurements

The absorption spectra were recorded using a Varian Cary 5000 UV–vis–NIR spectrophotometer. The samples were prepared by diluting NC samples in 3 mL of toluene in 1 cm path length quartz cuvettes with airtight screw caps in a N_2_ filled glovebox. The steady-state and time-resolved PL measurements were carried out on a Edinburgh FLS900 fluorescence spectrometer equipped with a Xe lamp and a monochromator for steady-state PL excitation and a time-correlated single photon counting unit coupled with a Edinburgh Instruments EPL-510 pulsed laser diode (λ_ex_ = 508.2 nm, pulse width = 177.0 ps) for time-resolved PL. The PLQY was measured using the IR 140 reference dye (95%, Aldrich) dispersed in DMSO, and all NC solutions were diluted to an optical density of 0.2 ± 0.04 at the excitation wavelength to minimize the reabsorption.

### Computational Methodology

To prepare InAs NC models, we cut the InAs zinc-blende bulk structure with symmetry *Fm*3*m* along the (111) facets and then we cleft the top of each vertex by removing InCl_3_ units. The final NCs have edges 2.2 nm long, in good agreement with the pure core InAs synthesized in the experiments. Structural relaxation calculations were carried out at the DFT/SCAN/DZVP level of theory with CP2K 8.2. The relaxation of the model substantially preserves the zinc-blende phase. Both density of states (DOS) and inverse participation ratio (IPR, see below) were computed by performing a single point calculation on the relaxed structure used for the exchange–correlation term, a combination of the mTASK functional for the exchange part and the PW92 for the correlation energy. In order to identify any surface localized state that could trap charge carriers, we also computed the IPR, which has been demonstrated to be a valid tool to identify traps in other NCs.^40^ The IPR quantifies the orbital localization of a given molecular orbital, and it is defined as
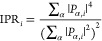
Here, *P*_α*i*_ represents the weight of molecular orbital *i* on a given atom α expanded in an atomic orbital basis. For finite systems, the IPR provides an estimate of the number of atoms that contribute to a given molecular orbital *i*. It can range from 0, a situation in which the wave function is distributed equally over all atoms in the system (i.e., a delocalized state), to 1, in the case of states localized on single atoms.

## Results and Discussion

We first optimized the synthesis of InAs NCs, starting from the work of Srivastava et al.,^32^ by systematically varying the reaction temperature (from 240 to 300 °C), the injection temperature of amino-As and DMEA-AlH_3_ (from 150 to 240 °C), and the reaction time (from 5 to 30 min) while keeping the other reaction parameters fixed (5 mL of oleylamine, InCl_3_:amino-As:DMEA-AlH_3_ precursors molar ratio of 1:1:3; see the Experimental Section and Figure S1 of the Supporting Information). The best parameters were chosen by checking the absorption curves of the resulting products and, in particular, the HWHM of the first excitonic absorption peak.^35,36^ Our results indicated that the narrowest size distribution could be achieved when injecting the amino-As and DMEA-AlH_3_ at 240 °C and growing the NCs for 15 min at 240 °C (Figure S1).

**Figure 1:**
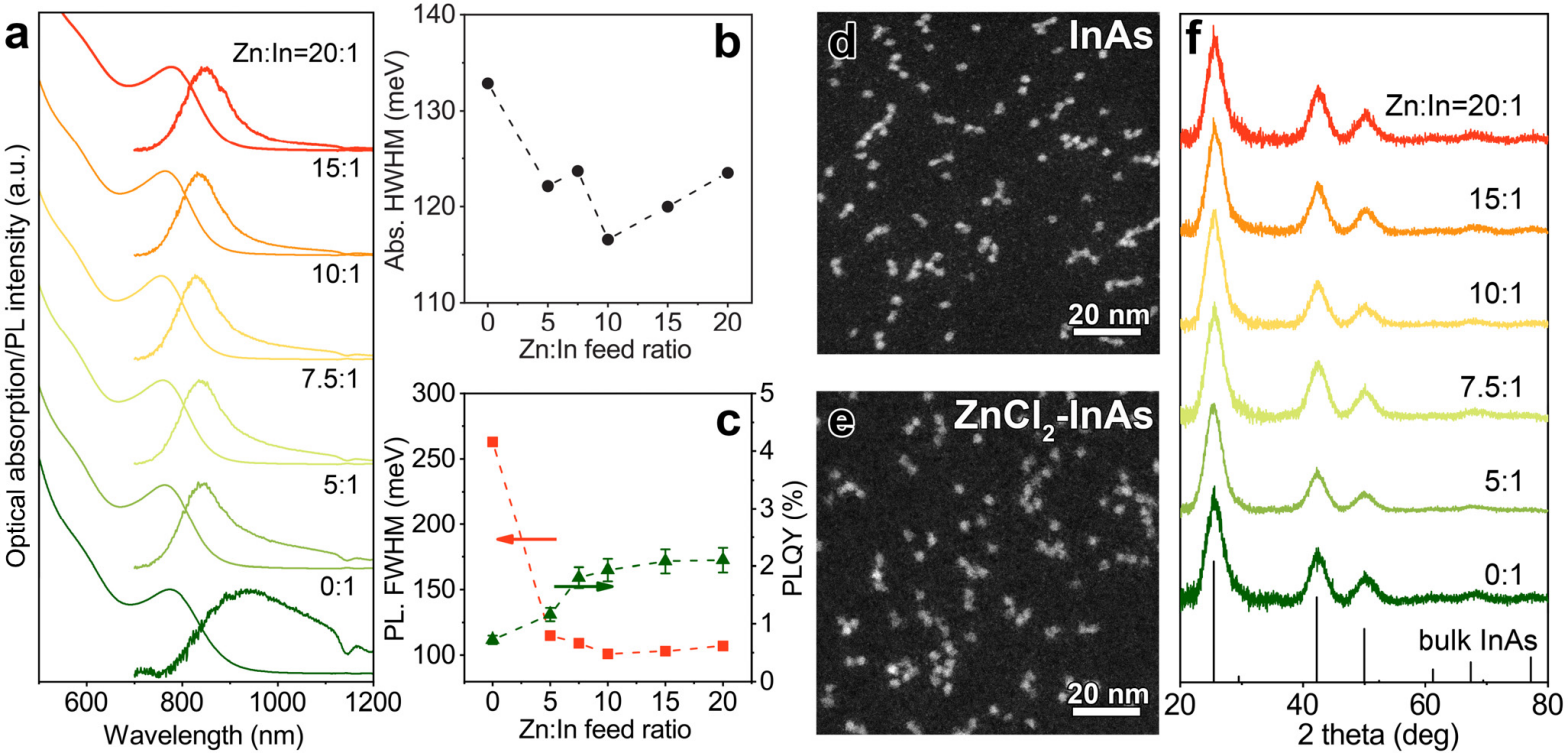
InAs NCs synthesized by different Zn:In feed ratios: (a) optical absorption and PL spectra; (b) HWHM of first excitonic peak; (c) full-width at half-maximum (FWHM) of PL spectra and PL quantum yield (QY); (d, e) representative HAADF-STEM images; (f) XRD patterns with the corresponding reflection of bulk zinc blende InAs (ICSD number 24518).

We then employed such optimized reaction parameters and added variable amounts of ZnCl_2_ (Zn:In ranging from 0:1 to 20:1) to the synthesis of InAs NCs. TEM (Figure S2) and STEM images of InAs NCs (Figure 1d,e) indicated that the products consisted of small particles with a narrow size distribution (∼2.8 ± 0.2 nm, Figure S3). The optical properties of these NCs are reported in Figure 1a. All the samples exhibited a clear excitonic absorption peak, whose HWHM was observed to decrease when employing ZnCl_2_ in the synthesis (Figure 1b). No shift of the excitonic peak occurred when varying the ZnCl_2_ amount (Figure 1a). On the other hand, whereas InAs NCs prepared in the absence of Zn did show a broad and weak PL emission, those synthesized with ZnCl_2_ were characterized by a more intense and narrower PL peak (Figure 1a,c). We also tried, as a control experiment, to run the same synthesis scheme in the presence of Zn(acetate)_2_, ZnBr_2_, or ZnI_2_ salts, but the quality of the NC product was very poor in terms of size control and of optical properties (Figure S4). Overall, our optical measurements indicated that ZnCl_2_ had a beneficial effect not only on yielding NCs with a narrower size distribution but also on improving the optical properties and luminescence efficiency of InAs NCs.

To understand the role of ZnCl_2_, we first evaluated the elemental composition of InAs NCs by performing ICP-OES, SEM-EDX, and XPS analyses (Table 1 and Table S1). The In/As ratio in all the samples was slightly higher than 1, pointing to NCs having an In-rich surface. The Cl/In ratio was around 0.4, indicating, in agreement with the work of Leemans et al.,^41^ that the excess of surface In atoms is passivated by Cl ions to maintain the overall charge balance (and available In surface sites are further passivated by neutral oleylammine). The amount of Zn increased from 0% to ∼8% when incrementing the Zn:In feed ratio from 0:1 to 20:1, indicating that Zn could be either inside the NCs (in the form of In–Zn–As alloy)^42^ or on their surface.

**Table 1:** Composition of InAs NCs Synthesized with Different Zn:In Feed Ratios

	InAs NCs elemental composition		
Zn:In feed ratio	In/As	Cl/In	Zn/In
0:1	1.12	0.36	0
5:1	1.35	0.39	0.04
7.5:1	1.22	0.42	0.05
10:1	1.14	0.42	0.06
15:1	1.10	0.46	0.07
20:1	1.18	0.44	0.08

With the aim of locating such Zn atoms, we first performed a X-ray diffraction (XRD) analysis which revealed that all the samples exhibited the expected InAs cubic zinc-blende crystal structure (Figure 1f). Importantly, no shift of the XRD peaks was observed when varying the amount of ZnCl_2_, which suggested that, differently from what was previously observed for InP NCs (where small Zn additions caused a marked change in lattice parameter),^42^ no incorporation of Zn inside the InAs NCs lattice had occurred in the present case. Indeed, it has been reported that the use of Zn in the synthesis of InP NCs can lead to the formation of In(Zn)P alloy NCs. These are characterized by XRD peaks shifting to higher diffraction angles when increasing the amount of Zn incorporated.^42,43^ In order to understand if, in the present case, the Zn atoms are preferentially located on the surface of the NCs, a ligand stripping procedure with Et_3_OBF_4_ was performed which is known to be a mild stripping agent, hence not causing severe NCs degradation (see the Experimental Section for details)_._^37^ The elemental analysis of the NCs after the stripping procedure indicated no substantial variation in the In/As elemental ratio, while the amount of Zn was observed to strongly diminish, reaching a final constant value of ∼1.3–1.9% for all the samples (Table S2). Moreover, the optical analysis of the stripped NCs did not show any obvious shift in the NCs absorption, indicating that no variation in the NCs size had occurred (i.e., no removal of In and As atoms, Figure S5). Overall, our ligand stripping experiment suggests that Zn is located mainly on the surface of the InAs NCs (hence it is preferentially removed by the stripping procedure) and most likely is in the form of ZnCl_2_, acting as a Z-type ligand.

**Figure 2:**
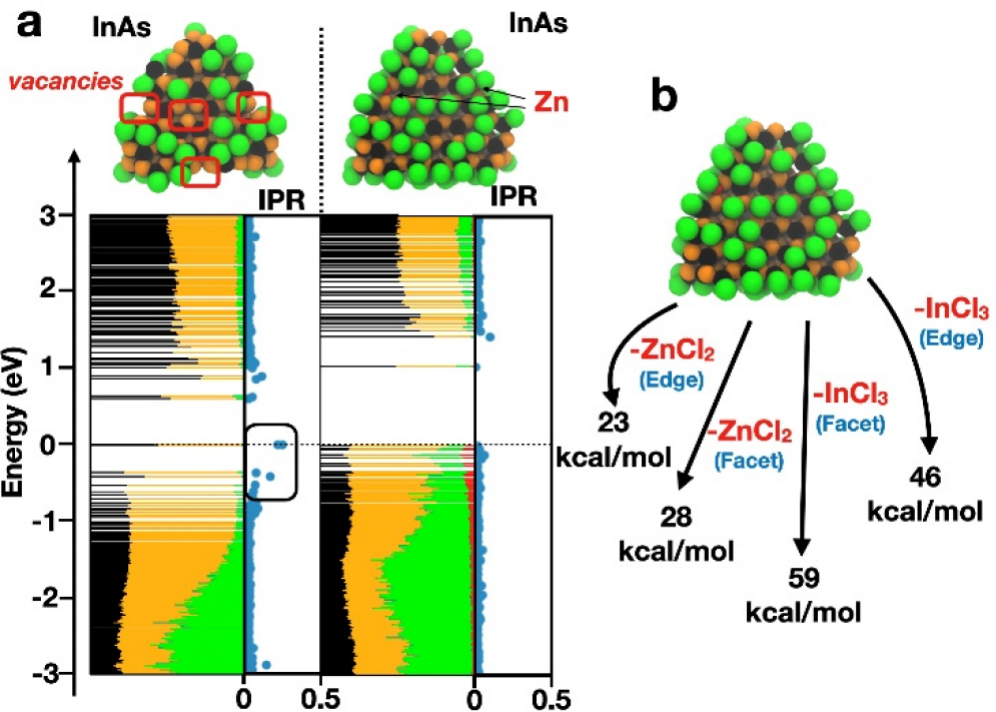
(a) Density of states computed at the DFT/mTASK/DZVP level of theory projected on each atom type (In, black; As, orange; Cl, green; Zn, red) for the InAs NC models with stoichiometry (InAs)_116_(InCl_3_)_17_ (left) and (InAs)_116_(InCl_3_)_25_(ZnCl_2_)_8_ (right). IPRs are also computed at the same level of theory. Values close to zero indicate full delocalization, while values close to 1 indicate localization on a single atom. (b) Computed binding energies for Z-type InCl_3_ and ZnCl_2_ moieties detached either from the center of the triangular (111) facets or from the edges.

To corroborate such hypothesis and to gain further insights on the surface of InAs NCs, we performed density functional theory (DFT) calculations. From the compositional analysis in Table 1, it was noted that by increasing the Zn/In feed ratio, the In/As ratio remained mostly unchanged, whereas the content of Cl increased. It was thus speculated that in the absence of Zn and assuming a charge balanced system, the InAs NCs present a significant number of In surface vacancies, which are filled (at least partially) by ZnCl_2_ when the latter is employed in the reaction mixture. On the basis of this assumption, we computed the electronic structure of two InAs NC models as depicted in Figure 2a. For simplicity, we did not include oleylamine molecules in the models because they would render the calculations more demanding. The model on the left represents a InAs NC made without ZnCl_2_ and features a (InAs)_116_(InCl_3_)_17_ stoichiometry (where the In/As and Cl/In ratios are 1.15 and 0.4, respectively). This nomenclature was used to highlight the excess of InCl_3_ present on the surface of the NCs. The model on the right has a (InAs)_116_(InCl_3_)_25_(ZnCl_2_)_8_ stoichiometry with 5% Zn (where the In/As and Cl/In ratios are 1.2 and 0.6, respectively). In both models, the compositions agree with the experimental results (Table 1). The first model (the one with no Zn) is characterized by several surface vacancies (In vacancies). Also, the valence band edge states have an inverse participation ratio (IPR) that deviates significantly from 0. This means that these states tend to be localized and possibly act as hole traps. On the other hand, the InAs model with 5% Zn has IPR values near zero, indicating an improved wave function delocalization also near the band edges. These results suggest that InAs NCs tend to have surface traps (and thus a broad and weak emission) that can be (partially) passivated by ZnCl_2_ Z-type ligands. This passivation in turn leads to the observed sharper emission lines and slightly improved PLQY (Figure 1a). However, it is not clear why ZnCl_2_ leads also to a better size distribution. The most plausible options are that (i) acting as a Z-type ligand, it might play a role in regulating the access of monomers to the facets of InAs NCs and (ii) it could interact with the amino-As precursor, as it does for the corresponding amino-P one,^30,31^ varying the reaction kinetics.

To assess the characteristics of the ZnCl_2_-InAs, the binding energy of the ZnCl_2_ moiety in different locations on the InAs surface (Figure 2b) was also computed. We noted that the binding of ZnCl_2_ along the edge of the NC is about 23 kcal/mol, whereas at the center of the triangular (111) facets the binding energy is higher (28 kcal/mol). Considering that the binding of InCl_3_ is much stronger (59 kcal/mol at the center of a 111 facet and 46 kcal/mol at the edge, Figure 2b), we can suggest the following: the treatment with Et_3_OBF_4_ can remove the ZnCl_2_ units mostly from the edges and partly from the center of the facets. This is in line with our previously discussed experimental observations that a fraction of ZnCl_2_ is still present on the NCs after the stripping procedure (Table S2). Interestingly, InCl_3_ remains well bound to the surface and energetically costly to be detached. Overall, our analyses revealed that the use of ZnCl_2_ in the synthesis of InAs NCs leads to an increased control over the size distribution and to a better surface passivation, due to the presence of ZnCl_2_ acting as Z-type ligands. Notably, the same beneficial effects cannot be achieved by simply replacing ZnCl_2_ with extra InCl_3_ in the synthesis approach or by the post-treatment of presynthesized InAs NCs with ZnCl_2_ (Figure S6), further indicating the importance of ZnCl_2_ as an additive in the synthesis itself.

We then proceeded with growing a shell of a wider bandgap material onto our InAs NCs with the aim of improving their PL. Among the most employed Cd-free wide-bandgap materials, we selected ZnSe, as this is one of those with the lowest lattice mismatch (although still as high as 6%) with InAs. As a reminder, the lattice parameters of InAs, ZnSe, and ZnS are 6.05–6.06 Å, 5.58–5.69 Å, and 5.35–5.43 Å, respectively.^25,30,31^ By taking advantage of the presence of an excess of Zn precursor in our synthesis approach, we devised a simple *in situ* strategy to grow the ZnSe shell (Scheme 1): the TOP-Se precursor was directly injected into the crude InAs NCs reaction mixture at 90 °C followed by heating up the overall solution to 300 °C for 15 min.

**Figure 3:**
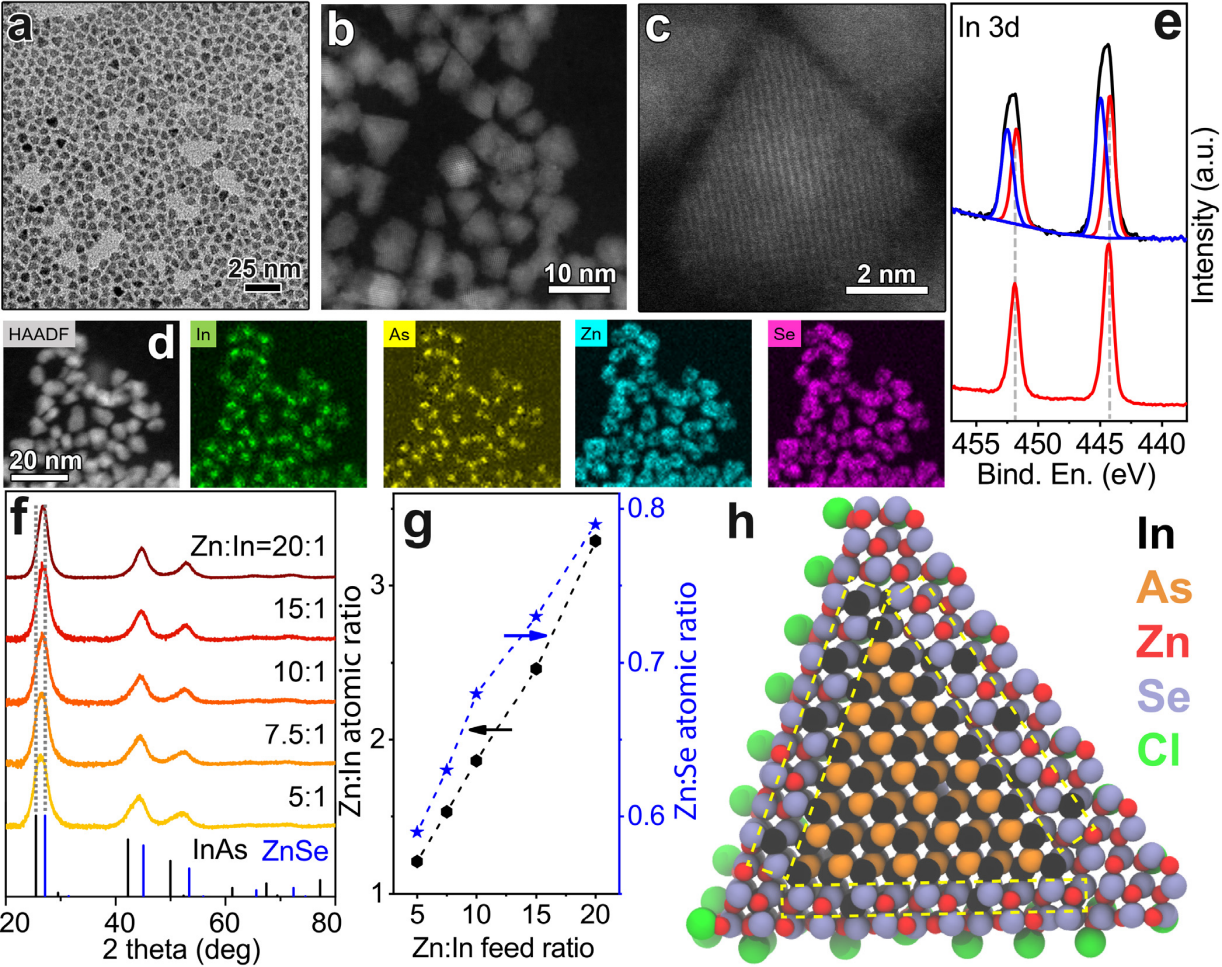
(a) TEM, (b) STEM, and (c) HRSTEM images of InAs@ZnSe core@shell NCs made from a Zn:In feed ratio of 20:1 with the corresponding (d) EDX elemental maps. (e) In 3d XPS spectra of InAs core (red curve) and InAs@ZnSe core@shell (black curve) NCs made with a Zn:In feed ratio of 20:1. InAs@ZnSe core@shell NCs obtained from InAs cores made with different Zn:In feed ratios: (f) XRD patterns with the corresponding reflection of bulk InAs (ICSD number 24518) and ZnSe (ICSD number 77092); (g) Zn:In and Zn:Se atomic ratios measured by SEM-EDX analysis. (h) Model of a InAs@ZnSe core@shell NC in which the hybrid In–Zn–Se layer between the pure InAs core and pure ZnSe outer shell is highlighted (in yellow). The model features In/As = 2.05, Zn/Se = 0.77, Zn/In = 2.16, Cl/Zn = 0.14 atomic ratios, in agreement with experimental values achieved for the sample made using a Zn:In feed ratio of 20:1 (Table 2).

The XRD patterns of the products were compatible with those of a cubic zinc-blende crystal structure having lattice parameters between those of InAs and ZnSe (Figure 3f), providing a first indication of the successful overgrowth of a ZnSe shell onto the InAs NCs. In detail, the higher the amount of ZnCl_2_ present in the reaction mixture, the closer the diffraction peaks shifted toward the values expected for ZnSe (Figures 3f and S7). The elemental analyses of these samples, performed via ICP-OES and SEM-EDX, further pointed to the successful formation of a ZnSe shell (Table 2 and Table S3). In detail, higher Zn/In and Zn/Se elemental ratios were measured for the samples prepared with greater amounts of ZnCl_2_ (Figure 3g and Table 2 and Table S3), even though the Se:In feed ratio employed in all the cases was set to 5:1. The combination of the elemental and XRD analyses therefore indicated that thicker ZnSe shells were produced when increasing the amount of ZnCl_2_ precursor.

**Table 2:** Composition of InAs@ZnSe Core@shell NCs Synthesized with Different Zn:In Feed Ratios

	InAs@ZnSe elemental ratio			
Zn:In feed ratio	In/As	Zn/Se	Zn/In	Cl/Zn
0:1	2.09	0.58	1.37	0.14
5:1	2.09	0.59	1.21	0.17
7.5:1	2.19	0.63	1.53	0.15
10:1	1.98	0.68	1.86	0.17
15:1	2.17	0.73	2.46	0.15
20:1	1.93	0.79	3.29	0.16

To further prove the formation of core@shell NCs, we performed a TEM analysis of the samples which indicated, in all the cases, an increase in the mean size (Figure S8). In particular, TEM and HAADF-STEM images of the NCs characterized by the thickest shell (i.e., highest Zn:In elemental ratio) showed that after the shell growth their size increased from 2.8 nm to ∼6.1 nm while retaining a narrow size distribution (±0.7 nm) (Figure 3a–c and Figure S9). High resolution HAADF-STEM images (Figure 3c), in combination with the elemental maps obtained via STEM-EDX (Figure 3d and Figures S10 and S11) indicated that (i) the final NCs are composed of a InAs core and a ZnSe shell region and (ii) the In map is slightly wider than that of As, but In is not found in the whole ZnSe shell, suggesting the presence of a In–Zn–Se interlayer (see also the discussion below).

Overall, our XRD, TEM, and elemental analyses confirmed the formation of core@shell NCs. Upon closer examination of the SEM-EDX results, an unexpected result was that the In/As elemental ratio in all the core@shell structures was around ∼2, that is, higher than the one found in the starting core NCs (i.e., ∼1.2, Tables 1 and 2), indicating that In was incorporated in the core@shell structures during the shell growth. Interestingly, such In/As ratio was independent from the thickness of the ZnSe shell, which ranged from ∼1 ML (corresponding to a Zn/In ratio of 1.2, observed in the sample prepared with a Zn:In feed ratio of 5:1) to ∼2 MLs (corresponding to a Zn/In ratio of 3.4, observed in the sample prepared with a Zn:In feed ratio of 20:1). This observation would exclude a simple migration of In from the core to the shell. It also indicates that In is not localized in the whole shell in the form of an In–Zn–Se alloy, as also evidenced by our STEM-EDX analyses of Figures S10 and S11; otherwise one would expect the amount of In to increase together with the number of shell layers.

To further elucidate this point, we analyzed in detail the XPS spectra of the InAs@ZnSe NCs (Figures 3e and S8). The data show the presence of Zn, Se, In, and As, with negligible oxygen content. Whereas the signals of As do not show appreciable differences from those of the InAs core NCs (in both cases, the As 3d_5/2_ peak is centered at 40.7 ± 0.2 eV, Figure S12, in agreement with reports on InAs^29,44^), the In 3d peaks observed in the core@shell sample are symptomatic of an inhomogeneous In environment. In fact, In 3d peaks observed on the InAs@ZnSe sample have a larger FWHM with respect to those observed on InAs cores (Figure 3e). This could be interpreted, according to our peak decomposition, as at least two different types of coordination (Figure 3e). We indeed identified signals that can be assigned to In(III) in InAs (In 3d_5/2_ at 444.2 ± 0.2 eV and In 3d_3/2_ at 451.8 ± 0.2 eV, as in the case of the core NCs) and another In 3d doublet with In 3d_5/2_ and In 3d_3/2_ components at (445.0 ± 0.2) eV and (452.6 ± 0.2) eV, respectively (Figure 3e and Figure S12). The position of such peak is close to that expected for In_2_Se_3_ (444.8 ± 0.3 eV), suggesting that part of In(III) cations in our systems are also coordinated with Se anions. In this context, we could exclude the formation of a In–Zn–Se alloyed shell; otherwise the amount of In (Table 2) would have increased together with that of Zn and Se. Overall, our XPS analysis and STEM-EDX data suggest that In is localized only at the interface (i.e., in the first shell layer) between the InAs core and the pure ZnSe shell, thus forming an In–Zn–Se intermediate layer, analogous to what observed in similar InP@ZnSe systems.^45^ A schematic view of the core–shell system including the intermediate In–Zn–Se layer is provided in Figure 3h where we closely followed the XPS stoichiometric ratios to build the NC model.

**Figure 4:**
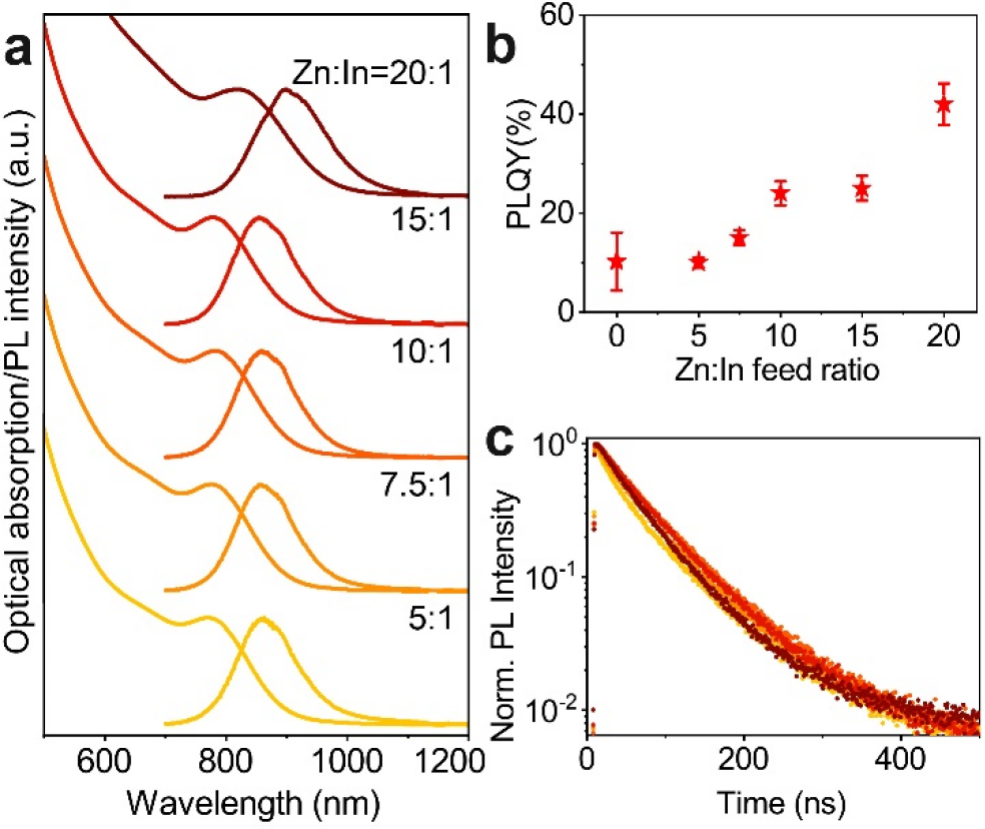
(a) Optical absorption and PL spectra, (b) PLQY values, and (c) PL decay curves of InAs@ZnSe core@shell NCs obtained from InAs cores made with different Zn:In feed ratios.

The optical absorption and PL spectra of the core@shell NCs are reported in Figure 4a. While all the samples exhibited a similar absorption exciton peak with HWHM ∼140 meV, a PL peaked at ∼860 nm with FWHM of 195 meV (Figure 4a and Table S4), and a PL lifetime in the range of 59–70 ns (Figure 4c and Table S5), the PL quantum yield (QY) was observed to increase together with the Zn:In feed ratio up to 42±4% (Figure 4b and Figures S13–S15). Such optical results are of particular interest considering that, independent from the As precursor employed, the lattice mismatch between InAs and common nontoxic shell materials such as ZnSe and ZnS typically leads to strained InAs@ZnX (X = S, Se) NCs with low PLQY values (maximum 10%).^25,30,31^ To attenuate such strain, complex architectures, in which intermediate shell layers are grown (resulting in core@multishell), have been fabricated, such as InAs@InZnP@GaP@ZnSe (PLQY of 23% and synthesized via TMGe-As).^26^ We believe that, in our case, the presence of an In–Zn–Se layer at the interface of the InAs@ZnSe core@shell NCs (emerging naturally from our synthesis) helps to reduce such lattice strain, therefore leading to an improved PLQY.

## Conclusion

We have synthesized InAs NCs via a hot-injection approach relying on the use of amino-As, as the As precursor, and DMEA-AlH_3_, as the reducing agent, in the presence of variable amounts of ZnCl_2_, employed as an additive. The use of ZnCl_2_ was observed to bring improvements in the InAs NCs size distribution and PLQY. Our DFT calculations indicated that ZnCl_2_ species can act as Z-type ligands capable of passivating surface traps, identified as In vacancies. Moreover, the presence of ZnCl_2_ in our reaction environment allows for the *in situ* overgrowth of a ZnSe shell on top of the InAs cores. The so-obtained InAs@ZnSe core@shell NCs exhibit PLQY values as high as 42±4% (with emission at ∼860 nm). Such high values were ascribed to the formation of an In–Zn–Se layer between the InAs core and the ZnSe shell, which might reduce the lattice strain at the interface of the two materials. Our procedure, which provides the brightest core@shell structures achievable with the amino-As precursor, represents a new starting point to further improve the synthesis of amino-As based InAs NCs by exploring, for example, different core@graded-shell or core@shell@shell architectures.

## Supporting Information Available

Optimization of the reaction parameters of InAs NCs; TEM images, size distribution histograms, optical properties, and elemental analyses of InAs and InAs@ZnSe NCs; magnification of XRD patterns, HWHM of the first exciton absorption peak, FWHM of the PL peak, and PL decay parameters of InAs@ZnSe core@shell NCs; synthesis and characterization of InAs@ZnSe core@shell NCs made from InAs NCs prepared in the absence of ZnCl_2_; XPS spectra of ZnCl_2_–InAs and InAs@ZnSe core@shell NCs made with a Zn:In feed ratio of 20:1 (PDF)
