# Supplementary material for: ZnCl2 Mediated Synthesis of InAs Nanocrystals with Aminoarsine
Source: J Am Chem Soc. 2022 Jun 1;144(23):10515–23. doi: 10.1021/jacs.2c02994 (PMC9204758; doi:10.1021/jacs.2c02994)
Supplement: Supplementary file 1 — ja2c02994_si_001.pdf [file ja2c02994_si_001.pdf]

Supporting Information for:

$ZnCl_2$ Mediated Synthesis of InAs Nanocrystals with Aminoarsine

Dongxu Zhu[a], Fulvio Bellato[a,c], Houman Bahmani Jalali[b] , Francesco Di Stasio[b], Mirko Prato[d], Yurii P. Ivanov[e], Giorgio Divitini[e], Ivan Infante[a], Luca De Trizio*[a], Liberato Manna*[a]

[a] Nanochemistry, [b] Photonic Nanomaterials, [d] Materials Characterization and [e] Electron Spectroscopy and Nanoscopy, Istituto Italiano di Tecnologia, Via Morego 30, 16163 Genova, Italy

[c] Dipartimento di Chimica e Chimica Industriale, Università di Genova, 16146 Genova, Italy

Optimization of the reaction parameters of InAs nanocrystals.

In a typical synthesis, 0.2 mmol of $InCl_3$ and 5 ml of oleylamine were loaded into a 100 ml three-necked flask under an inert atmosphere and dried at 120°C under vacuum for 1 hour. The mixture was heated to either 150°C (Figure S1b) or 240°C (Figure S1a,c,d). The As precursor (0.2 mmol of $As(NMe_2)_3$ dissolved in 0.5 ml dry oleylamine) was then injected into the flask, quickly followed by the injection of 1.2 ml of the DMEA-AlH_3 toluene solution. After the injection, the reaction temperature was set to either 240°C (Figure S1a-b), 260°C (Figure S1c) or 300°C (Figure S1d). The reaction was carried out for 30min, taking aliquotes after 5, 10 and 15 min. The NCs were washed by the addition of toluene and ethanol and precipitated by centrifugation at 4500 rpm for 5 min. The precipitate was dispersed in toluene and centrifuged at 5500 rpm for 5 min to remove the insoluble byproducts. The supernatant was precipitated by the addition of ethanol followed by centrifugation at 4500 rpm for 5 min. The optical density and the half-width at half-maximum (HWHM) of the first exciton absorption peak of the final products are reported in Figure S1.

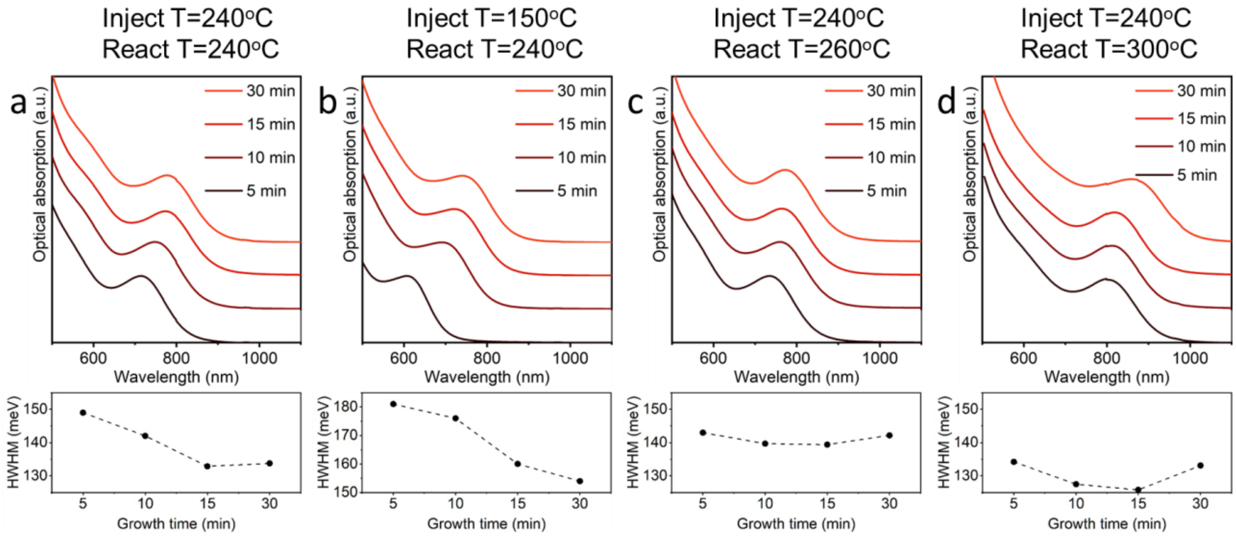

Figure S1. Optical absorption and corresponding HWHM of the first exciton absorption peak of InAs NCs obtained by injecting the Amino-As precursor and DMEA-AlH_3 reducing agent at either 150°C (b) or 240°C (a,c,d) and by running the reaction at 240°C (a,b), 260°C (c) or 300°C (d). All the other reaction parameters were not varied: 5ml oleylamine, 0.2mmol of $InCl_3$, $InCl_3$:amino-As:DMEA-AlH_3 precursors molar ratio of 1:1:3

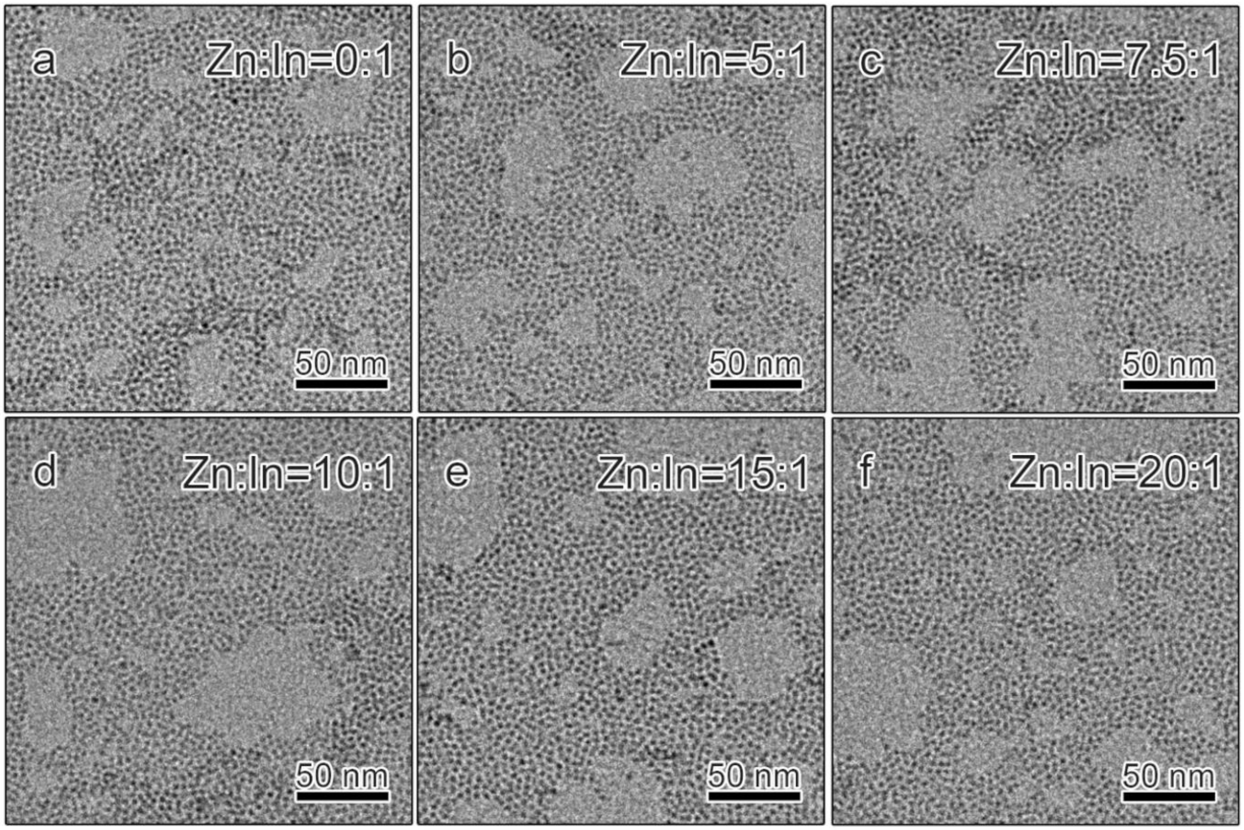

Figure S2. TEM images of $ZnCl_2$-InAs NCs produced with different Zn:In feed ratios.

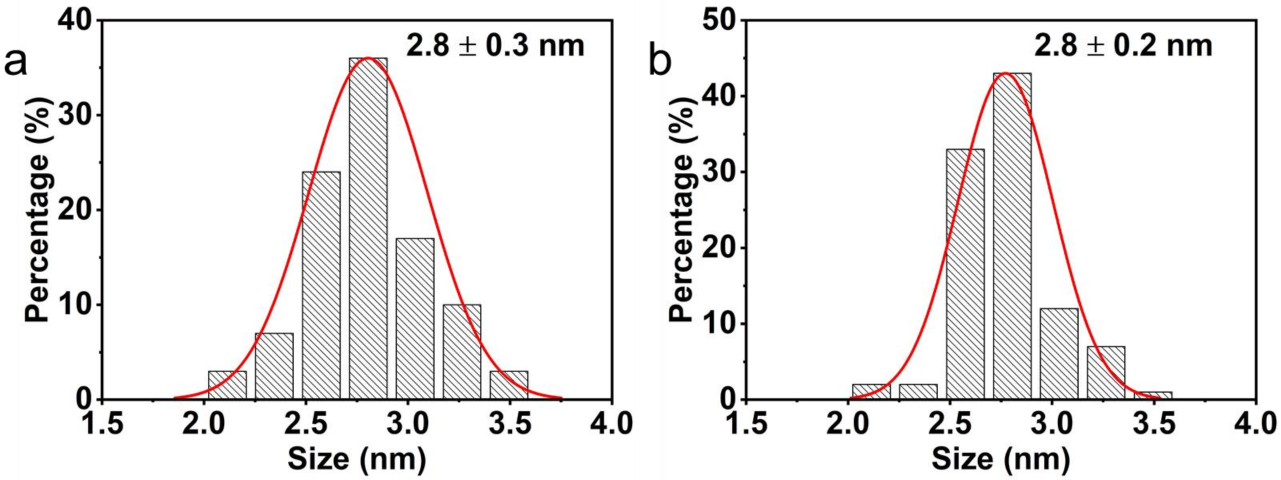

Figure S3. Size distribution histogram obtained from STEM images of (a) InAs NCs and (b) $ZnCl_2$-InAs NCs produced with Zn:In=10:1 feed ratio.

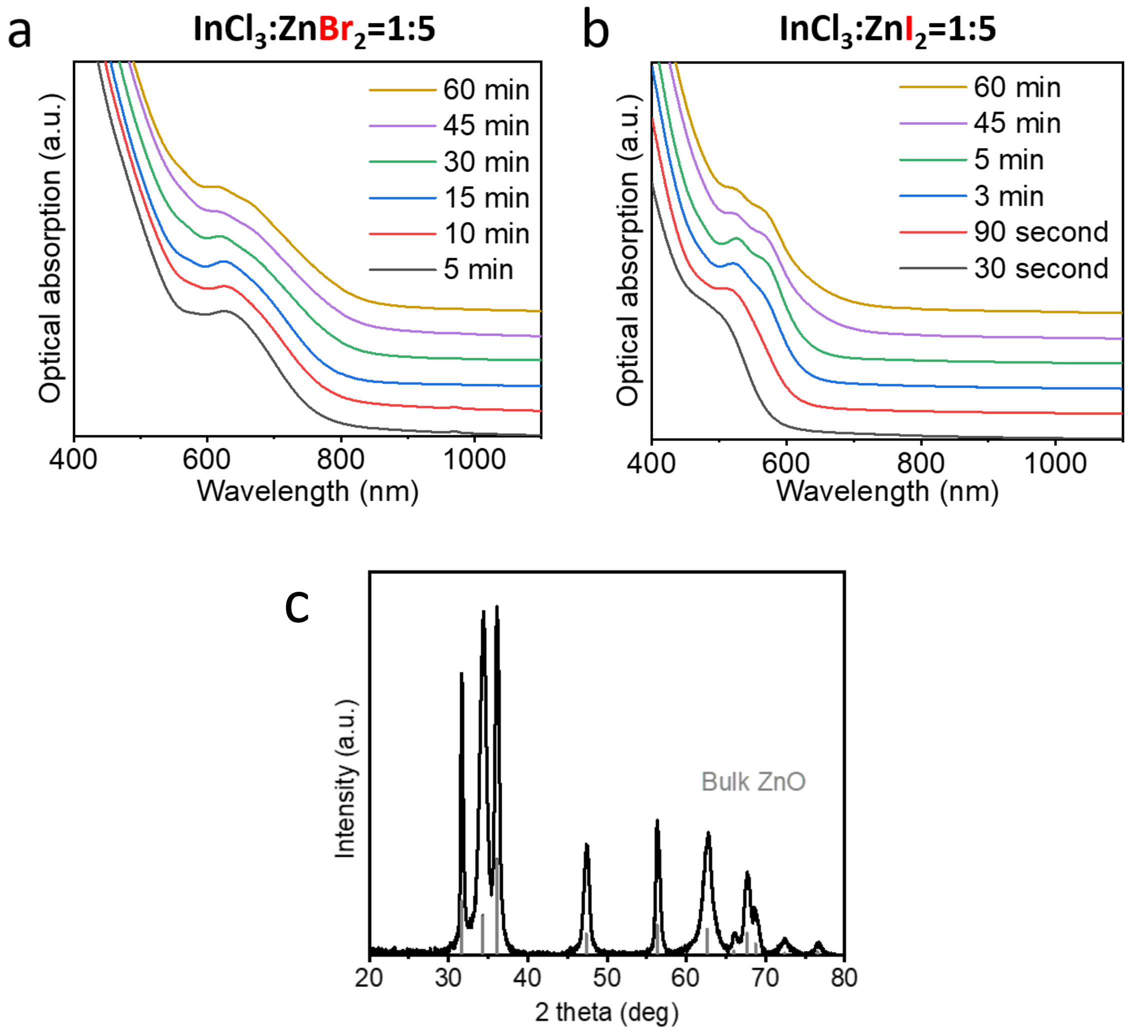

Figure S4. Optical absorption spectra of ZnX_2-InAs NCs obtained using a Zn:In feed ratio of 5:1 with different Zn halide sources: (a) $ZnBr_2$, (b) ZnI_2. (c) XRD pattern of $Zn(ac)_2$-InAs NCs obtained using a Zn:In feed ratio of 5:1.

Table S1. SEM-EDX, XPS and ICP-OES elemental analyses of InAs NCs synthesized with different Zn:In feed ratios

Zn:In feed ratio	SEM-EDX		XPS			ICP-OES
	In/As	Cl/In	In/As	Zn/In	Cl/In	Zn/In
0:1	1.12	0.36	1.18	0	0.21	0
5:1	1.35	0.39	-	-	-	0.04
7.5:1	1.22	0.42	-	-	-	0.05
10:1	1.14	0.42	1.21	6.9%	0.20	0.06
15:1	1.10	0.46	-	-	-	0.07
20:1	1.18	0.44	1.22	9.7%	0.19	0.08

Table S2. SEM-EDX, XPS and ICP-OES elemental analyses of InAs NCs after performing the ligand stripping procedure with EtO_3BF_4

Zn:In feed ratio	SEM-EDX		XPS			ICP-OES
	In/As	Cl/In	In/As	Zn/In	Cl/In	Zn/In
0:1	1.09	0.31	1.30	0	0.20	0
5:1	-	-	-	-	-	0.013
7.5:1	-	-	-	-	-	0.015
10:1	1.04	0.34	1.31	1.5%	0.19	0.019
15:1	-	-	-	-	-	0.017
20:1	1.18	0.31	1.25	1.8%	0.21	0.019

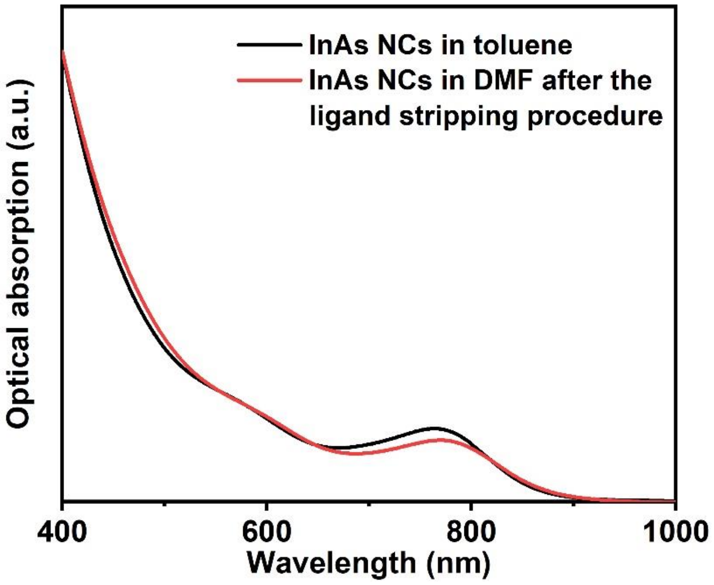

Figure S5. Optical absorption spectra of $ZnCl_2$-InAs NCs obtained using a Zn:In feed ratio of 15:1 before and after the ligand stripping treatment with EtO_3BF_4.

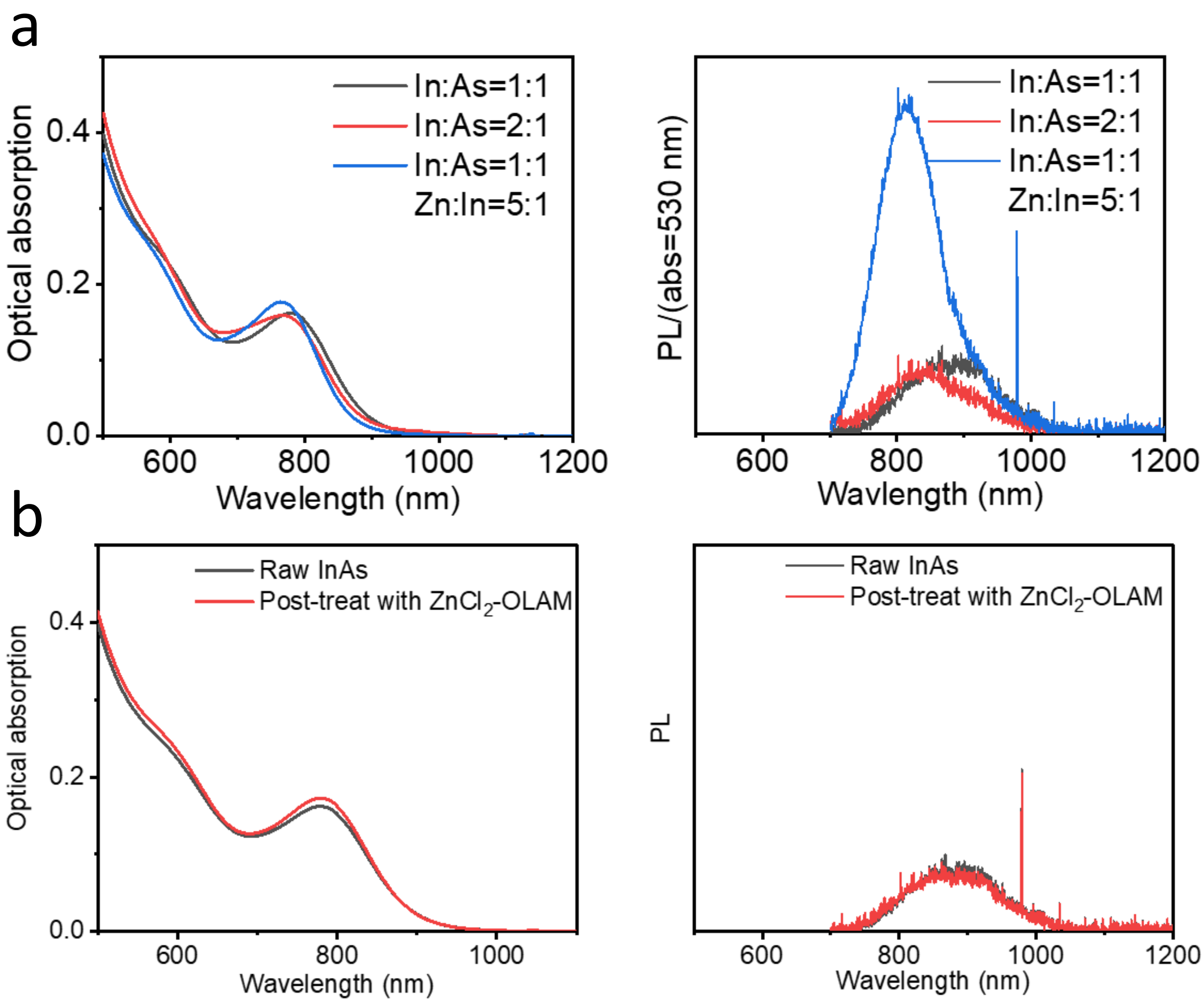

Figure S6. (a) Absorption curves (left) and PL (right) of InAs NCs made with In:As ratios of 1:1 (black curves) and 2:1 (red curves) in the absence of $ZnCl_2$. The PL of InAs NCs made with In:As=1:1 and Zn:In=5:1 is also shown for comparison (blue curve). (b) Absorption curves (left) and PL (right) of InAs NCs made with In:As ratio of 1:1 before (black curves) and after (red curves) the post-synthesis treatment with ZnCl2 (dissolved in oleylamine).

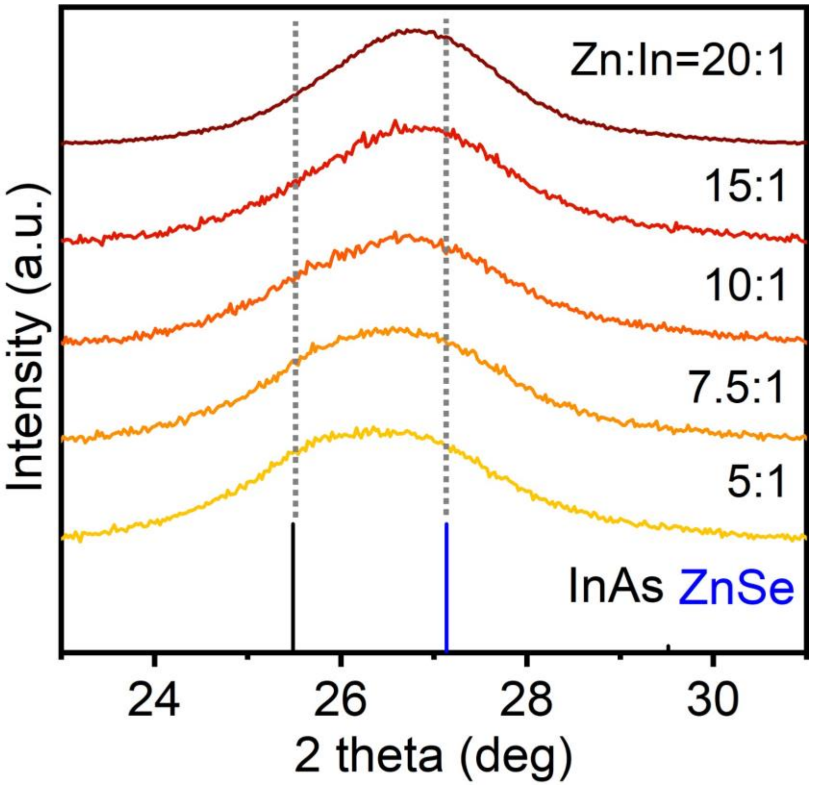

Figure S7. Magnification of the XRD patterns of InAs@ZnSe core@shell NCs, obtained from InAs cores made with different Zn:In feed ratios, in 23-31° the range.

Table S3. SEM-EDX and ICP-OES elementl analyses of InAs@ZnSe core@shell NCs

Zn:In feed ratio to synthesize the InAs core		SEM-EDS					ICP-OES		
		In/As	Zn/Se	Zn/In	Cl/In	Cl/Zn	In/As	Zn/Se	Zn/In
0:1	5:1*	2.09	0.58	1.37	0.20	0.14	1.87	0.75	0.99
	20:1*	2.35	0.66	2.06	0.29	0.14	2.11	0.88	1.94
	20:1**	2.23	0.69	2.47	0.37	0.15	2.15	0.88	2.45
1:5		2.09	0.59	1.21	0.21	0.17	1.82	0.76	1.00
7.5:1		2.19	0.63	1.53	0.23	0.15	1.90	0.80	1.19
10:1		1.98	0.68	1.86	0.31	0.17	1.69	0.86	1.56
15:1		2.17	0.73	2.46	0.37	0.15	1.83	0.89	1.92
20:1		1.93	0.79	3.29	0.54	0.16	1.66	0.98	2.95

* The synthesis of the ZnSe shell is based on the addition of a $ZnCl_2$-OLAM solution before the injection of the TOP-Se precursor (see the "Synthesis of InAs@ZnSe core@shell NCs starting from InAs NCs prepared in the absence of $ZnCl_2$" paragraph below)

**The synthesis of the ZnSe shell is based on the addition of $ZnCl_2$ powder before the injection of the TOP-Se precursor (see the "Synthesis of InAs@ZnSe core@shell NCs starting from InAs NCs prepared in the absence of $ZnCl_2$" paragraph below)

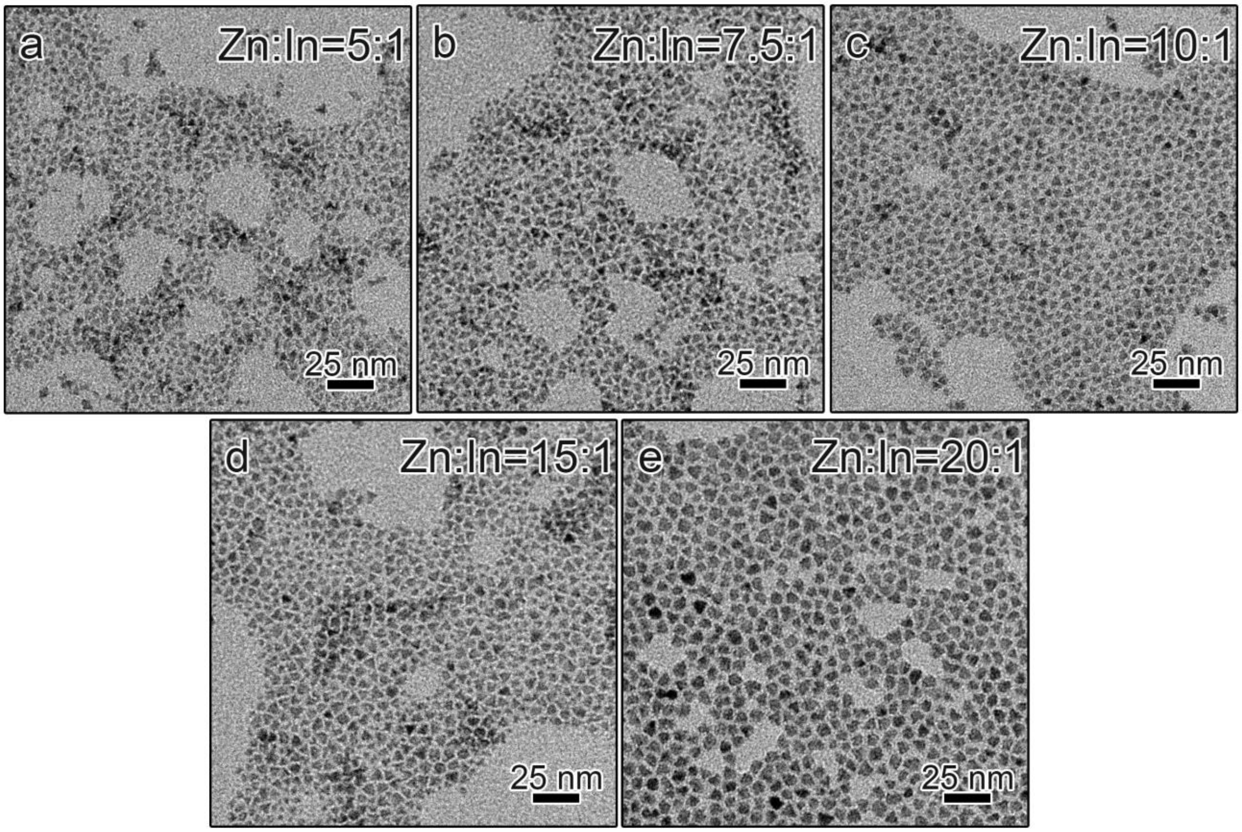

Figure S8. TEM images of InAs@ZnSe core@shell NCs synthesized with variable Zn:In feed ratios: (a) 5:1, (b) 7.5:1, (c) 10:1, (d) 15:1, (e) 20:1.

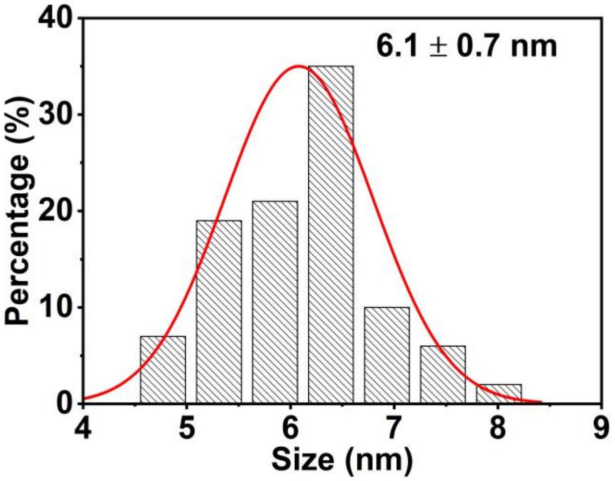

Figure S9. Size distribution histogram obtained from STEM images of InAs@ZnSe core@shell NCs synthesized with a Zn:In feed ratio of 20:1.

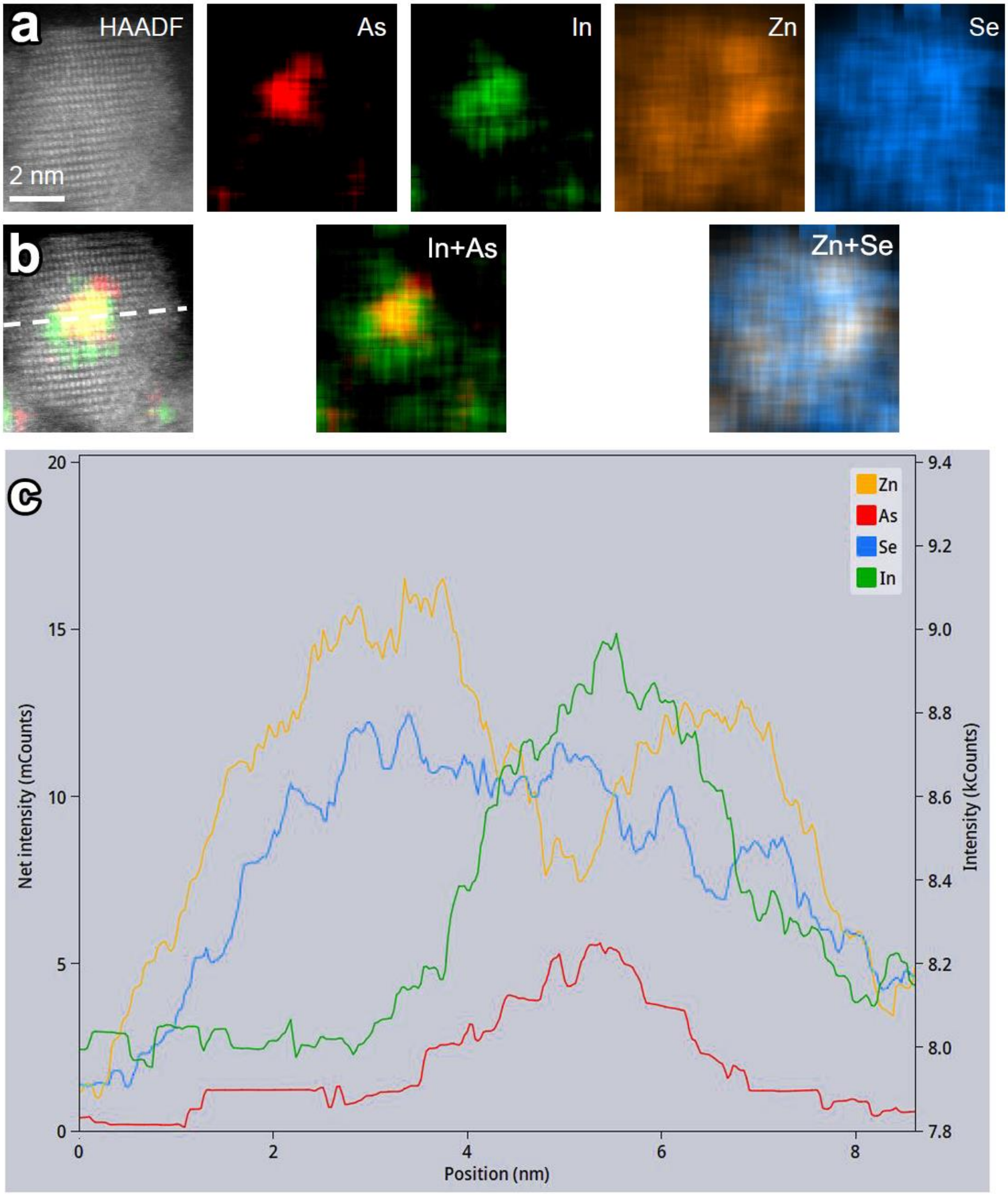

Figure S10. (a) HAADF-STEM image of InAs@ZnSe core@shell NCs made from a Zn:In feed ratio of 20:1 with the corresponding EDX elemental maps. (b) HAADF-STEM image of the crystal in (a) with the InAs core highlighted in colors and the corresponding EDX linescan (c).

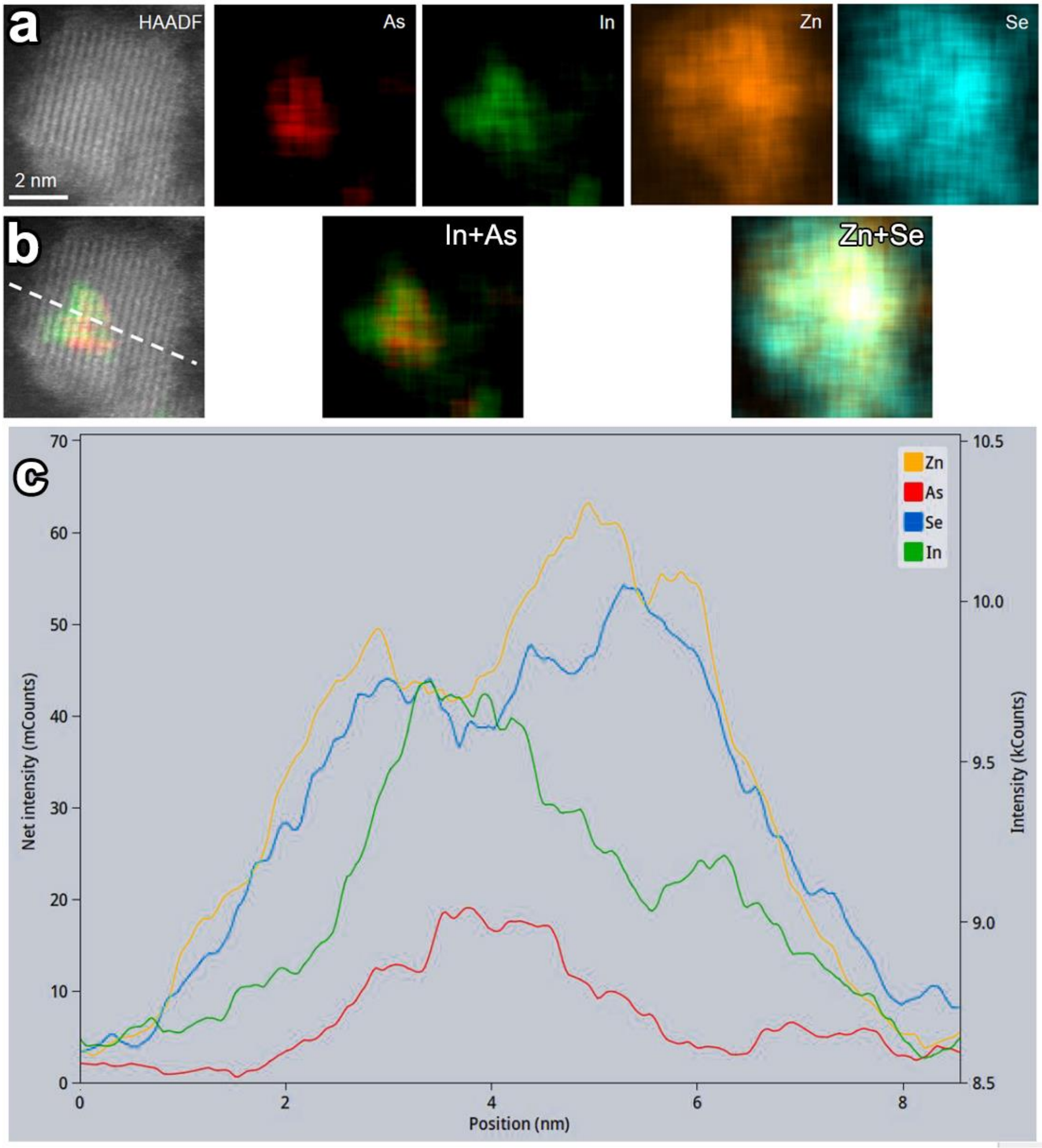

Figure S11. (a) HAADF-STEM image of InAs@ZnSe core@shell NCs made from a Zn:In feed ratio of 20:1 with the corresponding EDX elemental maps. (b) HAADF-STEM image of the crystal in (a) with the InAs core highlighted in colors and the corresponding EDX linescan (c).

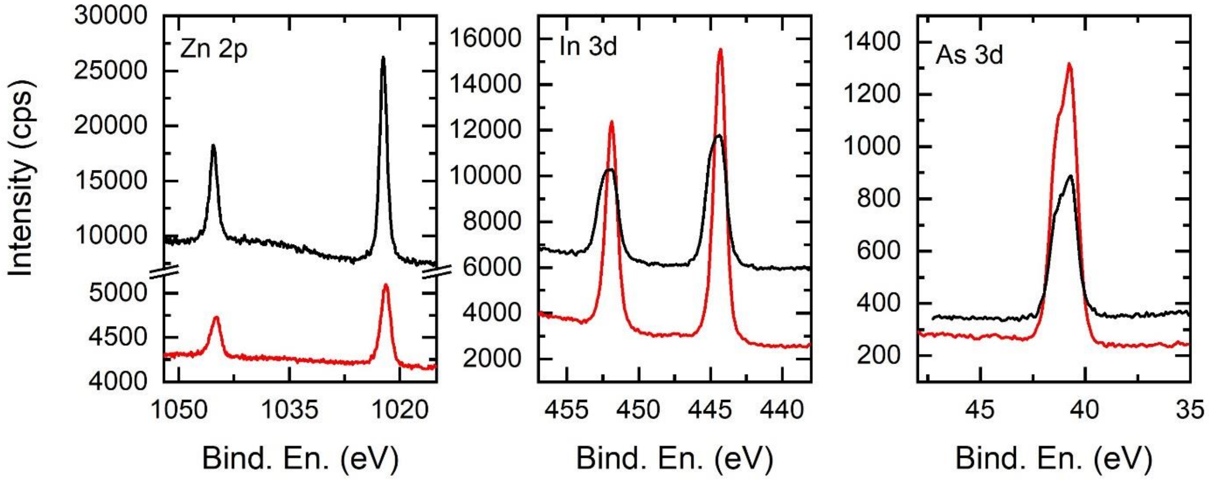

Figure S12. Zn 2p, In 3d and As 3d spectra of $ZnCl_2$-InAs NCs obtained with a Zn:In feed ratio of 20:1 (red curves) and corresponding core@shell NCs (black curves).

Table S4. HWHM of the first exciton absorption peak and FWHM of the PL peak of InAs@ZnSe core@shell NCs

Zn:In feed ratio to synthesize the InAs core	Abs. HWHM (meV)	PL FWHM (meV)
5:1	147	190
7.5:1	137	188
10:1	139	191
15:1	138	197
20:1	149	200

Table S5. PL decay parameters of InAs@ZnSe core@shell NCs

Zn:In feed ratio to synthesize the InAs core	A	B_1	τ_1(ns)	B_2	τ_2(ns)	τ_{ave}(ns)
5:1	0.01	0.44	21.20	0.55	71.95	62.26
7.5:1	0.01	0.42	29.25	0.58	75.99	65.81
10:1	0.01	0.50	44.37	0.49	82.97	69.38
15:1	0.01	0.59	45.25	0.40	85.46	67.91
20:1	0.01	0.62	40.43	0.38	75.26	58.94

The decay was fitted with a bi-exponential decay function:

$Y = A+B_1*\exp(-t/\tau_1) +B_2*\exp(-t/\tau_2)$

B_i and τ_i are amplitudes and PL lifetime. The average lifetime (τ_{ave}) was calculated by the function:

$\tau_{ave} = (B_1* \tau_1^2 + B_2* \tau_2^2)/(B_1* \tau_1 + B_2* \tau_2)$

Synthesis of InAs@ZnSe core@shell NCs starting from InAs NCs prepared in the absence of $ZnCl_2$

InAs NCs were synthesized employing 0.2 mmol of $InCl_3$ and 5 ml of oleylamine which were loaded into a 100 ml three-necked flask under an inert atmosphere and dried at 120°C under vacuum for 1 hour. The mixture was heated to 240°C and the As precursor (0.2 mmol of $As(NMe_2)_3$ dissolved in 0.5 ml dry oleylamine) was then injected into the flask, quickly followed by the injection of 1.2 ml of the DMEA-AlH_3 toluene solution. The reaction was carried out for 15 min and was quenched by removing the heating mantle. When the temperature reached 90°C, we followed three different procedure to grow the ZnSe shell:

i) 1.25 ml of 0.8 M $ZnCl_2$-OLAM solution (Zn/In ratio of 5:1) was added to the reaction flask followed by the injection of 1 ml of 1M TOP-Se (1 mmol of Se, Se:In ratio of 5:1) and the resulting mixture was heated up (heating rate ~30°C/min) to 300°C for 15 min. The characterization of the resulting NCs is shown in Figure S8. To prepare the 0.8 M $ZnCl_2$-OLAM solution, 8 mmol of $ZnCl_2$ and 10 ml oleylamine were loaded into a 100 ml three-necked flask under N_2 and degassed at 120 °C under vacuum for 1 hour. The final solution was transferred and stored into a nitrogen filled glovebox. The 0.8 M $ZnCl_2$-OLAM solution solidifies at room temperature, so it has to be preheated before use.

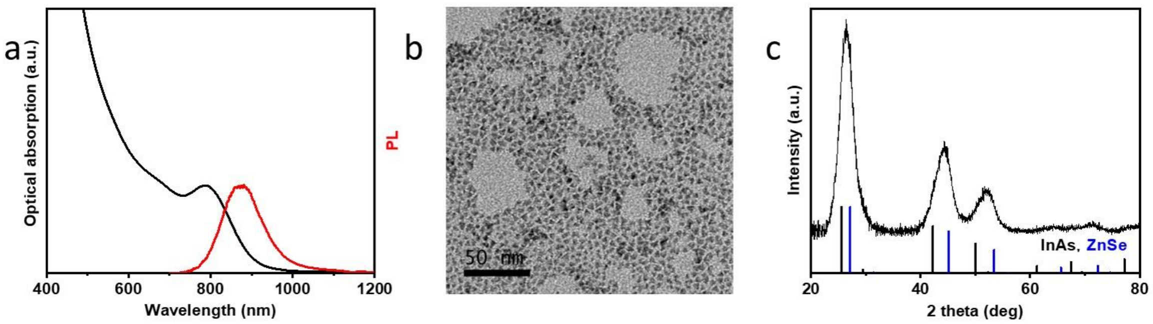

Figure S13. Optical properties (a), TEM image (b) and XRD pattern (c) of InAs@ZnSe core@shell structures synthesized starting from InAs NCs prepared in the absence of $ZnCl_2$, and by growing the ZnSe via the addition of 1.25 ml of 0.8 M $ZnCl_2$-OLAM solution (Zn/In ratio of 5:1). PLQY=12%.

ii) 5 ml of 0.8 M $ZnCl_2$-OLAM solution (Zn:In ratio of 20:1) was added to the reaction flask followed by the injection of 1 ml of 1M TOP-Se (1 mmol of Se, Se:In ratio of 5:1) and the resulting mixture was heated up (heating rate ~30°C/min) to 300°C for 15 min. The characterization of the resulting NCs is shown in Figure S9.

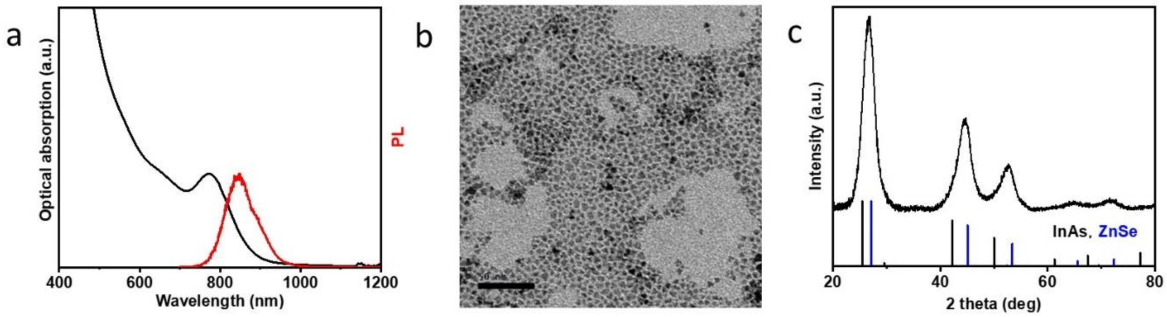

Figure S14. Optical properties (a), TEM image (b) and XRD pattern (c) of InAs@ZnSe core@shell structures synthesized starting from InAs NCs prepared in the absence of $ZnCl_2$, and by growing the ZnSe via the addition of 5 ml of 0.8 M $ZnCl_2$-OLAM solution (Zn/In ratio of 20:1). PLQY=17%.

iii) 4 mmol of $ZnCl_2$ powder (Zn/In ratio of 20:1) was added to the reaction flask followed by the injection of 1 ml of 1M TOP-Se (1 mmol of Se, Se:In ratio of 5:1) and the resulting mixture was heated up (heating rate ~30°C/min) to 300°C for 15 min. The characterization of the resulting NCs is shown in Figure S10.

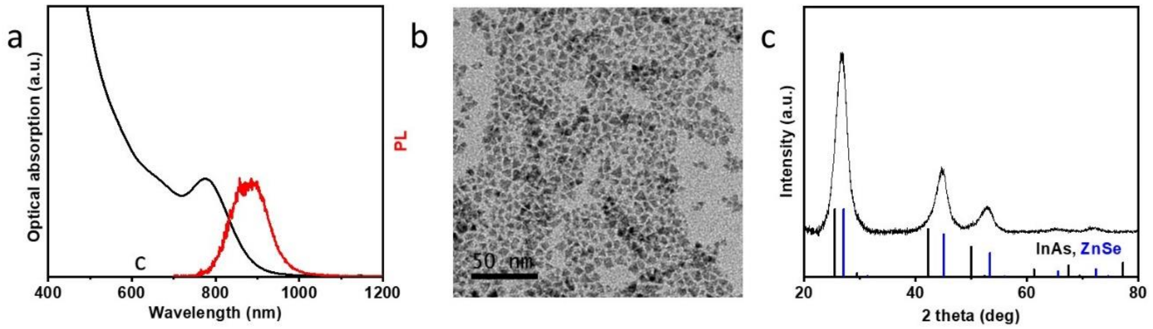

Figure S15. Optical properties (a), TEM image (b) and XRD pattern (c) of InAs@ZnSe core@shell structures synthesized starting from InAs NCs prepared in the absence of $ZnCl_2$, and by growing the ZnSe via the addition of 4 mmol of $ZnCl_2$ powder (Zn/In ratio of 20:1). PLQY=2%.

The PLQY values measured for these 3 samples were used in the plot of Figure 4b (Zn:In feed ratio of 0)
